# Chronic respiratory diseases other than asthma in children: the COVID-19 tsunami

**DOI:** 10.1186/s13052-021-01155-9

**Published:** 2021-11-06

**Authors:** Maria Di Cicco, Maria Giulia Tozzi, Vincenzo Ragazzo, Diego Peroni, Ahmad Kantar

**Affiliations:** 1 Allergology Section, Paediatrics Unit, Pisa University Hospital, Via Roma n. 67, 56126 Pisa, Italy; 2 Department of Clinical and Experimental Medicine, University of Pisa, Via Roma n. 55, 56126 Pisa, Italy; 3 Paediatrics and Neonatology Division, Women’s and Children’s Health Department, Versilia Hospital, Via Aurelia n. 335, Lido Di Camaioree, Italy 55049; 4 Paediatric Asthma and Cough Centre, Istituti Ospedalieri Bergamaschi - Gruppo Ospedaliero San Donato, via Forlanini n. 15, 24036 Ponte S. Pietro - Bergamo, Italy; 5 Vita-Salute San Raffaele University, Via Olgettina n. 58, 20132 Milan, Italy

**Keywords:** Children, SARS-Cov-2, Telemedicine, Pediatric respiratory diseases, ACE-2

## Abstract

Coronavirus disease 2019 (COVID-19) affects all components of the respiratory system, including the neuromuscular breathing apparatus, conducting and respiratory airways, pulmonary vascular endothelium, and pulmonary blood flow. In contrast to other respiratory viruses, children have less severe symptoms when infected with severe acute respiratory syndrome coronavirus 2 (SARS-CoV-2). A minority of children experience a post-infectious inflammatory syndrome, the pathology and long-term outcomes of which are poorly understood. The reason for the lower burden of symptomatic disease in children is not yet clear, but several pathophysiological characteristics are postulated. The SARS-CoV-2 pandemic has brought distinct challenges to the care of children globally. Proper recommendations have been proposed for a range of non-asthmatic respiratory disorders in children, including primary ciliary dyskinesia and cystic fibrosis. These recommendations involve the continuation of the treatment during this period and ways to maintain stability. School closures, loss of follow-up visit attendance, and loss of other protective systems for children are the indirect outcomes of measures to mitigate the COVID-19 pandemic. Moreover, COVID-19 has reshaped the delivery of respiratory care in children, with non-urgent and elective procedures being postponed, and distancing imperatives have led to rapid scaling of telemedicine. The pandemic has seen an unprecedented reorientation in clinical trial research towards COVID-19 and a disruption in other trials worldwide, which will have long-lasting effects on medical science. In this narrative review, we sought to outline the most recent findings on the direct and indirect effects of SARS-CoV-2 pandemic on pediatric respiratory chronic diseases other than asthma, by critically revising the most recent literature on the subject.

## Introduction

In December 2019, China reported an increasing number of cases of severe pneumonia of unknown etiology in the city of Wuhan, the capital of Hubei Province [1]. Some weeks later, the causative agent was identified as a novel coronavirus, 2019-nCoV, which shows about 79% identity with SARS-CoV-1, the virus that caused a severe interstitial pneumonia epidemic in 2002–2004 in Asia [2]. 2019-nCoV was later named SARS-CoV-2, and its clinical manifestations were named COVID-19 (CoronaVirus Disease - 2019). In the next few weeks, the virus rapidly spread around the world, and the infection was declared as a pandemic by the World Health Organization in March 2020 [3]. The SARS-CoV-2 pandemic has caused more than 200 million of cases and 4 million deaths as of early August 2021, which has had a huge impact on the everyday lives of every human on Earth. The evidence thus far has shown higher mortality among males, the elderly, and those with underlying chronic conditions, such as diabetes, hypertension or ischemic heart disease [4, 5].

**Fig. 1:**
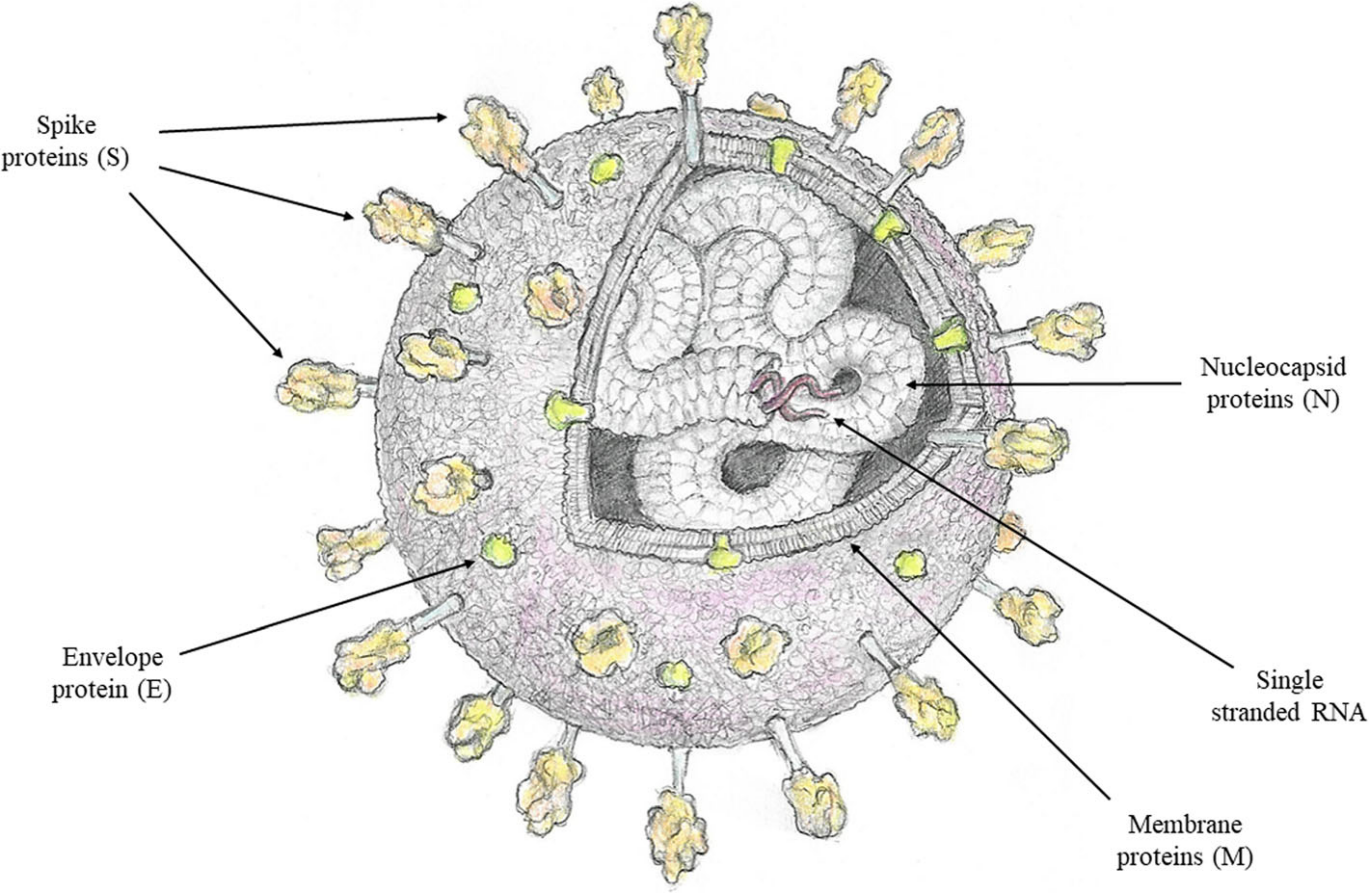
Structure of SARS-CoV-2 virion. SARS-CoV-2 belongs to the Coronaviridae family and contains a single stranded RNA genome encoding four structural proteins, namely spike (S), envelope (E), nucleocapsid (N) and membrane (M) proteins. S, E and M proteins constitute the viral coat, while N protein packages the viral genome. SARS-CoV-2 targets the host cells through S proteins, which bind to ACE-2 receptor. TMPRSS2, a type 2 transmembrane serine protease, is needed to activate S proteins for membrane fusion, enabling cell infection

In childhood, SARS-CoV-2 infection seems to be less common, and the disease is generally milder [6, 7]. However, several studies have shown that even when presenting with mild symptoms, children may be a source of contagion [8–10] and may complain of long-lasting symptoms. Although only a minority of children with COVID-19 require hospitalization, severe cases have been reported [11, 12]. Children with at least one pre-existing underlying medical condition are at higher risk for severe disease, but lower risk has been detected with increasing age [13–15]. Several hypotheses have been proposed to explain the generally milder course of the disease in children and adolescents (Table 1). One possibility is a different expression of angiotensin-converting enzyme 2 (ACE-2), which represents the SARS-CoV-2 receptor, and transmembrane protease serine 2 (TMPRSS2), which activates the structural spike protein of SARS-CoV-2 for membrane fusion and enables cell infection (Fig. 1). There is evidence of lower ACE-2 expression in the nasal epithelium in children than in adults [16], while higher ACE-2 expression has been found in adult smokers and those with chronic obstructive pulmonary disease (COPD), which have been associated with more severe disease [17, 18]. Moreover, children have a large thymic repertoire, sustained innate immunity, and more T and B regulatory lymphocytes than adults. Children experience several respiratory infections in the first years of life, including infections by other coronaviruses. These infections and the vaccinations that children receive might constitute a robust immunological stimulus that could activate the immune system and make it more efficient at containing pathogens in general. Due to this so-called “*trained immunity,*” children could have a more protective immune response than adults [6, 19, 20] The peculiarities of immune responses in children, together with the continuous maturation of the other bodily systems, could also determine differences in the clinical manifestation of COVID-19 between adult and pediatric patients: as an example, gastrointestinal symptoms, such as vomiting, abdominal pain, and diarrhea, are more commonly reported in children than in adults [21]. Moreover, “Multisystem Inflammatory Syndrome in Children related to COVID-19” (MIS-C) is a rare but severe complication that occurs 2–6 weeks after SARS-CoV-2 infection and is reported exclusively in predominantly previously healthy children and adolescents [22]. This life-threatening hyperinflammatory syndrome involves multiple organ systems and resembles Kawasaki disease (KD) but is a distinct entity. Compared to those with KD, children with MIS-C are older, have higher levels of systemic inflammation, myocardial injury and coagulopathy markers and more lymphocytopenia and thrombocytopenia [23]. Moreover, the course of the disease is usually worse, with higher complications and mortality rates.

**Table 1:** Proposed mechanisms explaining why children have a generally milder course of SARS-CoV-2 disease than adults

• Different ACE-2 expression or function in children than in adults (ref. 16)	
• Large thymic repertoire and sustained innate immunity (ref. 6, 21)	
• Elevated B, T and NK lymphocytes (ref. 6, 19)	
• Recurrent respiratory infections and vaccinations in the first years of life (trained immunity) (ref. 6, 19)	
• Reduced exposure to tobacco smoke (ref. 17)	
• Healthy respiratory system	
• Less comorbidities than adults

This paper discusses how children with chronic respiratory diseases (CRD) other than asthma have been affected by the SARS-CoV-2 pandemic at multiple levels and outlines recent data on the subject.

## Methods

To collect data for this narrative review, we searched relevant published studies in the MEDLINE/PubMed database by combining the following MeSH (Medical Subject Headings) terms and keywords: “SARS-CoV-2” or “COVID-19” or “Coronavirus Diseases 2019” AND “chronic lung diseases” or “chronic respiratory diseases” or “cystic fibrosis” or “primary ciliary dyskinesia” or “bronchopulmonary dysplasia” or “bronchiectasis” or “interstitial lung diseases”. The original search was run in December 2020 and updated in August 2021. The search strategy included filters for language (English) and age of study subjects (0–18 years) with no limit for the year of publication. Only papers published in peer-reviewed journals were reviewed, and we excluded studies that were not related to the subject of interest. Considering the shortage of data on children and adolescents, we chose not to exclude specific types of articles. The reference lists of the selected articles were fully and accurately scanned to identify potentially relevant studies that were not included during the first search. Two authors (MDC and AK) independently screened titles and abstracts and analyzed the full-text version of the selected papers.

## Are children with Chronic Respiratory Disease at Higher Risk for SARS-CoV-2 infection or severe COVID-19?

In the first months of the pandemic, a high number of severe COVID-19 cases or respiratory exacerbations was expected in adults and children with CRD. This was based on the consideration that their airways may be a potential *locus minoris resistentia* and that viral-induced exacerbations easily occur. Moreover, respiratory diseases affect hundreds of millions of people around the world, with almost 545 million CRD patients of all ages having been reported in 2017, who were mostly affected by COPD and asthma [24]. Nevertheless, CRD have not been identified as the most significant comorbidity for COVID-19 [25]. In fact, COPD and asthma appear to be underrepresented in these patients compared to their prevalence in the general population [26]. However, patients with underlying respiratory diseases who develop COVID-19 and are hospitalized have worse outcomes, and their case fatality rate is 6.3%, in contrast to the overall rate of 2.3% in China [25, 26]. In Italy, chronic lower respiratory diseases were the fifth most common comorbidity among 5311 deaths in adults related to COVID-19, which were behind hypertensive heart disease, diabetes mellitus, ischemic heart disease, and neoplasms [27]. Larger studies have found an association between COVID-19-related death in adults with severe asthma (defined as asthma with recent use of oral corticosteroids; odds ratio 1.13; 95% confidence interval [CI] 1.01–1.26), as well as in those with other respiratory diseases (odds ratio 1.63; 95% CI 1.55–1.71) [5]. Notably, a recent systematic review showed that patients with pre-existing COPD have more than 3 times higher risk of mortality and severe COVID-19 [28].

As far as children, a multicenter cohort study by Götzinger et al. reported on 582 cases of SARS-CoV-2 infection from 77 health-care institutions located in 21 European countries, comprising patients with a median age of 5.0 years (ranging from 3 days to 18 years). The study confirmed that COVID-19 is generally a mild disease in children, but a small proportion of children (8%) develop severe disease requiring support in the intensive care unit (ICU), including mechanical ventilation, often for 1 week or more. Furthermore, 25% of the study cohort patients had at least one pre-existing medical condition, and the most frequent were CRD (29 children, 16 with asthma, and 6 with bronchopulmonary dysplasia). The significant risk factors for ICU admission were found to be age less than 1 month, having at least one pre-existing medical condition, male sex, and the presence of lower respiratory tract infection signs or symptoms at presentation [15]. Moreover, a study by Bellino et al. included 3836 pediatric patients with a median age 11 years and reported on the Italian integrated COVID-19 surveillance system. The study included mild cases treated or isolated at home during the first wave of the pandemic. Of these patients, 13.3% were admitted to the hospital (of whom 3.5% were admitted to the ICU), and 4 deaths were reported, which all involved children with comorbidities. Overall, 5.4% of the study cohort had at least one pre-existing medical condition, and CRD were the most common, which is in agreement with the prevalence of chronic diseases in children. Furthermore, 4.3% had severe disease, and almost all of them were under 6 years of age. The authors found a higher risk of severe COVID-19 in patients with preexisting underlying medical conditions (odds ratio 2.80; 95% CI 1.74–4.48) and in children younger than 1 year of age [13]. However, it should be noted that both of these studies refer to the first wave of the pandemic, when the capacity of testing was much lower than nowadays, so that hospitalization and ICU admission rates may be falsely high. Nevertheless, a significantly larger study by Kompaniyets et al. reported data from a cross-sectional study that included more than 43.000 patients aged less than 18 years who were evaluated during an emergency department or inpatient encounter in the United States from March 2020 to January 2021, showing that 9.9% were hospitalized and 29.6% were admitted to the ICU [29]. 28.7% of the study cohort had one or more underlying medical condition, with asthma being the most common (10.2%). The study showed that chronic and complex chronic disease were risk factors for hospitalization (adjusted risk ratio [aRR] of 2.91; 95% CI 2.63–3.23 and 7.86; 95% CI 6.91–8.95 respectively) as well as for severe COVID-19 (aRR 1.95; 95% CI 1.69–2.26 and 2.86; 95% CI 2.47–3.32 respectively). The strongest risk factors for hospitalization were type 1 diabetes and obesity (aRR 4.60; 95% CI 3.91–5.42 and 3.07; 95% CI 2.66–3.54 respectively), while those for severe COVID-19 were type 1 diabetes (aRR 2.38; 95% CI 2.06–2.76) and cardiac and circulatory congenital anomalies (aRR 1.72; 95% CI 1.48–1.99). Prematurity was a risk factor for severe COVID-19 among children younger than 2 years (aRR 1.83; 95% CI 1.47–2.29) [29].

There is still limited data on SARS-CoV-2 infection in the context of respiratory diseases other than asthma and COPD, especially in children. It should be noted that in the paper by Kompaniyetes et al., 10.2% of patients had asthma, and 1.1% had other specified and unspecified upper respiratory diseases, while no other respiratory condition was reported to have a prevalence > 0.7% [29].

A COVID-19 survey involving the Paediatric Assembly of the European Respiratory Society (ERS) included data from 174 centers (80 centers reported no cases) and 945 children with COVID-19. The results showed that the infection was well tolerated among children with asthma and cystic fibrosis (CF), while a minority of children with bronchopulmonary dysplasia and other CRD required ventilatory support [30]. A recent study on almost 600 primary ciliary dyskinesia (PCD) patients including 219 subjects aged < 19 years found that only 2.1% of the study population tested positive for SARS-CoV-2 [25]. This result is comparable to that of 0.4% found in 7500 CF patients in another study carried out in France [26]. These two studies showed that COVID-19 caused more admissions to the hospital and ICU in those with CF than in PCD patients (61 and 13% vs. 8% and 0 respectively). However, it should be noted that the PCD study was based on an online questionnaire with voluntary enrollment, while the CF study was hospital-based.

Lastly, the first case series of SARS-CoV-2 infection in pediatric patients with CF including 105 children recently showed that most of the patients had a relatively mild illness, and those who were hospitalized had lower lung function and a lower body mass index [31]. To our knowledge, no data is available on COVID-19 in children with non-CF and non -PCD bronchiectasis or interstitial lung disease. In the case of the latter, studies on mortality in adults suggest caution, and longitudinal studies are needed to assess the risks for children on immunosuppressive agents [32].

Thus, it appears that both adults and children with CRD can catch the infection and develop symptoms with an overall increase in risk of severe COVID-19, which could be related to the underlying lung involvement degree. Nevertheless, after nearly two years of the pandemic, CRD continue to be underrepresented in the statistics of SARS-CoV-2 patients, and in case of CF, the incidence of COVID-19 has been reported to be lower than in the general population [33]. It has been speculated that this could be explained by the under-diagnosis of CRD in some countries in comparison to other chronic diseases, as well as by a deep commitment from patients and parents in practicing careful protective behaviors in order to avoid respiratory infections. In fact, these patients and their families are inevitably more prone to careful personal protection due to consolidated experience in hand hygiene, mask wearing, and social distancing [34–36]. Some authors suggest that CRD treatments may reduce the risk of infection or developing COVID-19, such as the chronic administration of azithromycin [37] and inhaled corticosteroids (ICS). Even if previous studies have linked ICS use to a possible increased incidence of upper respiratory tract infections and pneumonia due to an impairment of antiviral innate immune responses, some authors have recently speculated that ICS may interfere with SARS-CoV-2 infection and the natural course of COVID-19 by reducing ACE-2 expression [38]. Peters at al. confirmed that the use of ICS by those with asthma is dose-dependently associated with reduced ACE2 and TMPRSS2 gene expression, in contrast to systemic corticosteroids [39]. In that study, the expression of ACE2 and TMPRSS2 genes was analyzed in sputum cells in adults with asthma, which indicated no significant differences between asthma and healthy subjects. In spite of this evidence, higher risk of COVID-19-related death has been observed in COPD and asthma patients who were prescribed ICS than those on bronchodilators [40]. Consequently, we can speculate that it is not ICS that makes positive or negative differences in the natural course of the disease, but the underlying health differences between people prescribed ICS may be a factor (those on ICS are likely to have more severe disease). However, recent evidence has showed that inhaled budesonide may be beneficial in the early phase of the disease in terms of reduced likelihood of needing urgent medical care and reduced time to recovery [41, 42].

Overall, the evidence available so far does not suggest that changes in ICS treatment are needed in CRD patients [26, 43].

Last but not least, it should be noted that COVID-19 seems to have a more favorable outcome in children and adolescents with allergies due to higher eosinophil counts. The reason is that eosinophilic cationic protein and eosinophil-derived neurotoxin can effectively neutralize SARS-CoV-2 [19]. Interestingly, among adults who have died due to COVID-19, eosinopenia is very frequent [44]. Thus, it has been suggested to be a biomarker of poor prognosis and a predictor that could facilitate triage of adults with CODIV-19 [45]. There are limited data available for children indicating low eosinophil counts in SARS-CoV-2 patients [46]. Moreover, it should be noted that allergic sensitization is inversely related to ACE-2 expression [47].

## What impact is the pandemic having on pediatric respiratory disease management?

The pandemic has caused major changes in clinical practice worldwide at both the hospital and primary-care levels. In order to reduce the risk of spreading the infection between health care workers and patients, many outpatient and inpatient services were temporarily closed in the first wave of the pandemic, with only emergency care made available. During lockdown in Italy, outpatient visits to pediatric specialties were reduced by about 80%, which was also due to the parents’ fear of exposing their children to SARS-CoV-2 infection by attending healthcare facilities [48]. In the following months, many activities restarted, but with limitations. This has had unavoidable yet important implications that are still present in the management of chronic conditions, including respiratory ones, for which regular outpatient evaluation is of pivotal importance.

At the primary-care level, reduced or delayed medical evaluation is causing delays in the treatment of exacerbations and in the supply of drugs and medical devices. At the hospital and specialist levels, limited activity causes delays in CRD diagnosis, especially in cases of rare diseases, as well as reduced frequency and quality of follow-up visits due to the partial availability of routine assessments in response to the pandemic. It is not known what impacts there will be from deferring diagnostic and treatment procedures in these diseases, but we can expect an increase in exacerbations and admissions in the near future [49].

Nevertheless, the health care issues raised by the pandemic have pushed health care systems to rapidly improve and promote the use of telemedicine, which had already been available in many countries before the pandemic but was barely used up to 2020 [50, 51]. Telemedicine may be an easy and effective measure during viral outbreaks since it can reduce the need for patients to go into healthcare facilities while also guaranteeing the continuity of care [52]. In fact, many randomized controlled trials carried out before the pandemic have shown that telemedicine is feasible and at least not inferior to usual care for chronic patients, such as in COPD patients [53–55]. Moreover, web-based pulmonary rehabilitation seems to be as effective as in-person sessions for adult patients [56, 57]. Evidence on the effectiveness of telehealth during SARS-CoV-2 pandemic is starting to be available, with recent reports showing successful use of such techniques in many different chronic diseases including CF [58, 59]. Thus, the telemedicine approach could be improved and widely used for pediatric patients, especially adolescents, even in the post-pandemic era [60]. However, “*digital-divide*” issues could worsen disparities in health care. This should be taken into account, especially among those at lower socio-economic levels, who may not be able to acquire the appropriate technology or may not have internet access [49].

Children on home mechanical ventilation are a particularly high-risk group of patients for which telemedicine should be widely used to avoid hospital-related SARS-CoV-2 infection risk, as well as to continue patient care, including modification of the ventilator parameters when needed [61]. As a matter of fact, a recent study by Onofri et al. showed that the use of telemedicine in invasively or non-invasively ventilated children with medical complexity is a feasible tool to avoid in-person visits during pandemics. This would allow for the ventilator parameters to be changed and for patients to be monitored remotely [62]. One crucial point in respiratory care is that we are witnessing a steep fall in pulmonary function tests (PFT) due to the risk of person-to-person transmission of SARS-CoV-2, which mainly occurs via respiratory droplets or touching contaminated objects [63]. PFT are considered to be a risk factor for viral transmission due to the potential for coughing and droplet formation during the procedures, surface contamination, and close contact between the patient and PFT staff [64, 65]. As a consequence, in the first wave of the pandemic, only urgent PFT were performed to guide management for patents with CRD such as CF and PCD, severe uncontrolled asthma, or onco-haematological diseases.

Many adult and pediatric scientific societies have released recommendations regarding prevention and control strategies in PFT laboratories. Most of these recommendations agree on the need to limit PFT to selected cases to guide management and to follow strict infection control measures when PFT are performed [66]. Similarly, many different recommendations have been rapidly released for treatments in a wide range of CRD in both adults and children [43]. All of them agree in suggesting that children and adolescents with CRD should remain on their current medications, wear masks, practice physical distancing, and wash their hands frequently [67, 68].

## Will changes in lifestyles impact patients’ mental and physical health?

The SARS-CoV-2 pandemic has dramatically changed our lives in many different ways and has negatively impacted quality of life, which is particularly burdensome for people affected by chronic diseases. For these patients, the pandemic and the related protective measures to avoid the infection, such as social distancing, represent a risk for mental health. Children and adolescents are the most vulnerable subjects in having to face deep changes in daily routine and loneliness due to reduced social interactions, boredom, and fear of catching the infection. It is expected that children are becoming more irritable and hostile in this condition and that their adherence to treatments will be impacted negatively. Patients with CRD will be at particular risk considering that they may experience stigmas related to their respiratory symptoms such as chronic cough, as well as being marginalized by their peers [69].

One study surveyed 72 caregivers of children aged 7.3 ± 2.9 years with CF, asthma, tuberculosis, or allergic rhinitis. As expected, most of the children were stable in terms of physical health, but their mental health was deteriorated, and there was a significant increase in arguments with siblings and parents [70]. Moreover, 30.7% of the subjects skipped their regular check-ups due to the fear of catching the virus in healthcare facilities. This understandable concern is also causing vaccines and medical evaluations to be postponed, even in cases of exacerbations or medical emergencies in general [71]. These parents also fear that wearing masks will impact their children’s gas exchange, even though there is evidence that using facemasks is safe, even for elderly patients with COPD [72, 73].

Regarding lifestyle, in 2020, children’s physical activity was significantly reduced, while the hours spent in front of a television, smartphones, or tablets increased. At the same time, children are also eating less favorable diets due to the time spent at home, resulting in notable weight gain in many children worldwide. Such lifestyles have detrimental effects, especially in children with CRD, who benefit from healthy diets and physical activity in terms of aerobic fitness, quality of life, disease control, and lung function [74].

Moreover, staying at home for long periods causes a reduction in vitamin D production, which is particularly worrisome when considering the many effects that this vitamin has apart from ensuring optimal bone health [75]. In particular, vitamin D plays a pivotal role in the immune system response, and insufficient vitamin D levels increase the risk for infections in children with CRD. As a whole, changes in daily routine structure may also affect adherence and self-management among youth with chronic conditions [60], both negatively or positively depending on the subject and its family predisposition and habits. Another survey was carried out in Israel on 445 caregivers of patients aged 0 to 18 years with chronic respiratory disorders. Among these, the most common disorders were asthma (291 patients), recurrent pneumonia (96 patients) and bronchopulmonary dysplasia (32 patients). Notably, the results showed that during the first-wave lockdown, the clinical status worsened in about 10% of patients, who were more likely to be older than 5 years and to have increased screen time, decreased physical activity, and shorter sleep duration [76].

## What about research?

In the first months of 2020, the SARS-CoV-2 outbreak temporarily disrupted clinical trials worldwide [77]. In the first wave of the pandemic, healthcare facilities had to postpone or reduce outpatient activities, including those related to clinical trials, and many laboratory-based projects were temporarily suspended due to infection risks [78].

Patients started declining to go to the hospital, to continue investigative treatments, or to participate in new clinical trials. Many physicians and researchers were fairly committed to COVID-19 wards and SARS-CoV-2 research or had COVID-19 themselves. And as if that was not bad enough, there were interruptions in supply chains and monitoring of clinical trials [79]. Much funding has gone to COVID-19 management and prevention research [80]. Notwithstanding all these issues, research never stopped completely, including research on rare diseases, and scientists and sponsors are making incredible efforts to restore previous trials and starting new ones. At this stage, we cannot predict the long-term effects of all these issues on medical research, but we can reasonably expect at least delays in the development and marketing of new drugs.

## Future directions

In spite of a huge commitment of scientists, we still do not have a specific treatment for SARS-CoV-2 infection. Studies have shown a lack of efficacy for many of the approaches used so far, including hydroxychloroquine and several antivirals [81]. Nevertheless, systemic corticosteroids seem to slightly reduce all-cause mortality in adults hospitalized because of symptomatic COVID-19 [82] and several studies are ongoing on other promising drugs. In the meanwhile, there has been remarkable improvement in terms of ventilatory support and management of the complications in these patients. Scientists are racing to find the most efficient treatment approach for MIS-C in children [83].

Luckily, in 2021 the world has entered a new phase of the pandemic due to the approval of several SARS-CoV-2 vaccines, such as those based on mRNA and viral vectors technology [84]. As of early August 2021, about 31% of the world population has received at least one dose of a COVID-19 vaccine, and about 23% is fully vaccinated, but in low-income countries, only 1% of people have received at least one dose [85]. BNT162b2 (Pfizer; BioNTech) was the first COVID-19 vaccine to be approved in children aged > 12 years, then also Moderna’s mRNA-1273 was approved for those who are 12 years of age or older. Approval for mRNA vaccines administration from 6 years of age is being awaited for the next autumn, which will be of pivotal importance in achieving global herd immunity, preventing complications such as MIS-C, and reducing infection spread in children caused by more virulent variant strains that could potentially emerge [84]. Vaccination programs in high income countries have opened a phase of coexistence with SARS-CoV-2, allowing governments to start releasing people from mitigation measures, including in healthcare facilities. Normality is slowly returning, but future scenarios and new challenges are completely unknown.

Another subject for future studies will be the reported persistence of symptoms affecting different systems after the acute SARS-CoV-2 infection. These have recently been recognized as a distinct entity called “long covid” which could potentially occur in both people with mild and severe COVID-19. Symptoms include fatigue, dyspnea, cognitive impairment, sleep disturbance, muscle pain and headache [86]. Long covid is now also reported in childhood, but its potential impacts on chronic diseases are unknown [87, 88].

Last but not least, we believe we should also focus on the many different lessons that the pandemic has thought us, starting from the role of primary care medicine in facing outbreaks at a real-life population level. Moreover, mitigative measures to reduce the spreading of SARS-CoV-2 infection, including hand washing, mask wearing and social distancing, have significantly reduced admissions worldwide for children with and without chronic conditions, which has mainly been due to the reduction of circulation of all respiratory viruses [89, 90]. Notably, in 2020 patients with CF also benefited from the lockdown not only in terms of reduced exacerbations and hospital admissions, but also in improved respiratory function [91]. Such evidence should suggest that some of these measures could be still considered in the future to protect patients at higher risk. In particular, mask wearing (high-efficiency masks are now available worldwide and are socially accepted) and working or studying remotely could be advised for selected patients [92]. Moreover, the enhancement of the use of telemedicine is going to stay, and telehealth in general will play an increasing role in the care of patients with chronic conditions in the near future.

## Conclusions

**Fig. 2:**
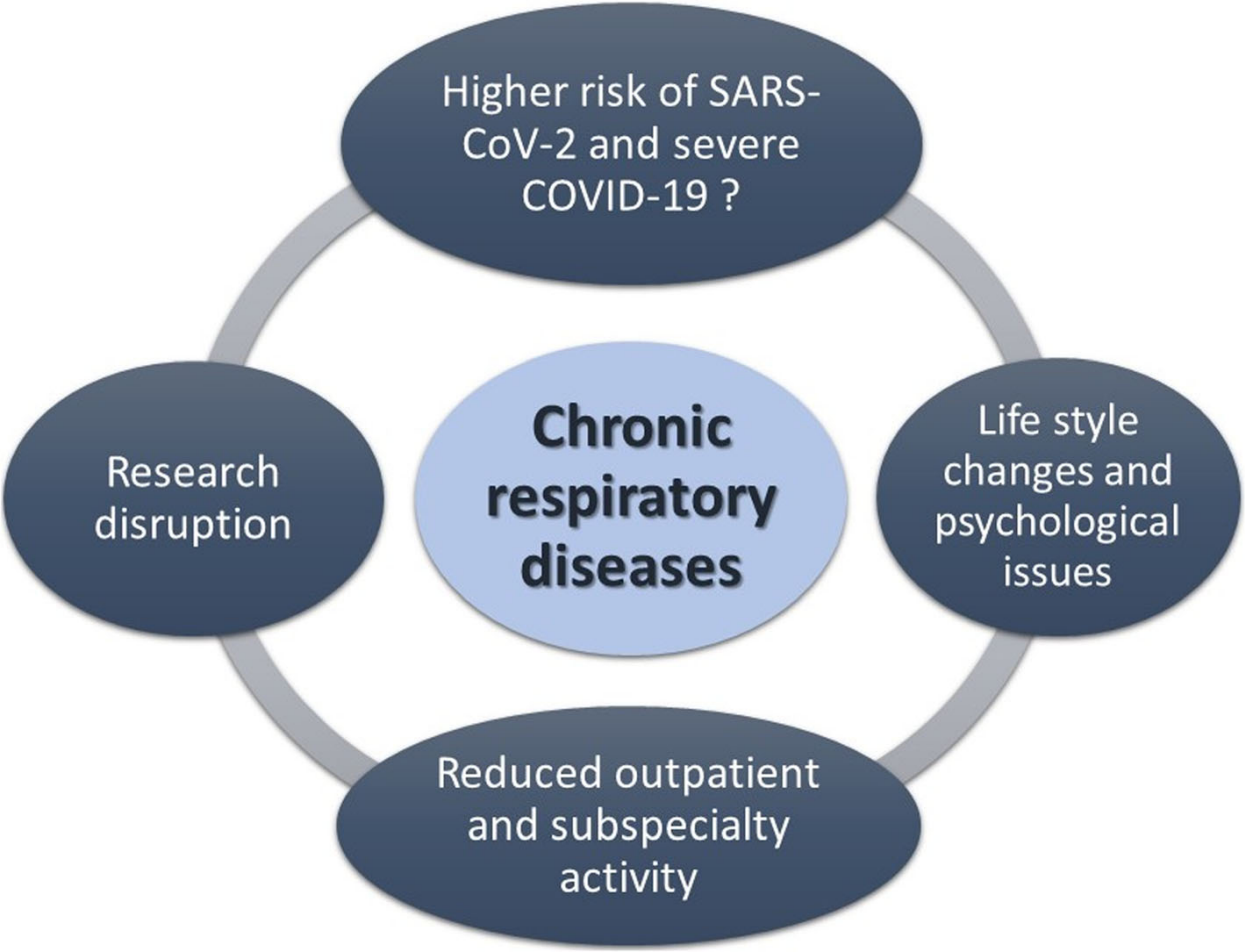
Impacts of COVID-19 pandemic on chronic respiratory diseases. The figure summarizes the main direct and indirect impacts of SARS-CoV-2 pandemic on chronic respiratory diseases which will have long-lasting effects which can only be imagined. Children will be at particular high risk and represent the most vulnerable group of patients

The world will not be the same when the SARS-CoV-2 pandemic ends. Hopefully, we will have learned many lessons, including how to build more efficient national health care systems that cooperate effectively in a worldwide network [25]. We believe that some of the mitigative measures will remain, such as wearing personal protective equipment in healthcare facilities or mask wearing among children at higher risk of severe respiratory infection. The incredible scientific run ending with SARS-CoV-2 vaccination is a milestone giving hope for future epidemics. The vaccines are already changing the course of the current pandemic and are now available also for children beyond 12 years old. Nevertheless, CRD patients will still have to face many issues directly or indirectly caused by SARS-CoV-2 (Fig. 2). We should encourage them and their families to continue to be cautious in order to avoid the infection, even when having completed their vaccination schedule, as well as continuing their treatments and follow-ups regularly and to seek immediate medical care when needed. Avoiding air pollution and smoke exposure, being physical active, and receiving other vaccinations should also be recommended in order to promote respiratory health even during a pandemic [93].

## Data Availability

Not applicable.

## Abbreviations

ACE-2: Angiotensin-Converting Enzyme - 2
aRR: adjusted Risk Ratio
CI: Confidence Interval
CF: Cystic Fibrosis
COPD: Chronic Obstructive Pulmonary Disease
COVID-19: CoronaVirus Disease - 2019
CRD: Chronic Respiratory Diseases
ICS: Inhaled CorticoSteroids
ICU: Intensive Care Unit
KD: Kawasaki Disease
MIS-C: Multisystem Inflammatory Syndrome in Children related to COVID-19
PCD: Primary Ciliary Dyskinesia
PFT: Pulmonary Function Testing
